# A Macroporous Magnesium Oxide-Templated Carbon Adsorbs Shiga Toxins and Type III Secretory Proteins in Enterohemorrhagic *Escherichia coli*, Which Attenuates Virulence

**DOI:** 10.3389/fmicb.2022.883689

**Published:** 2022-05-06

**Authors:** Hidetada Hirakawa, Kazutomo Suzue, Motoyuki Uchida, Ayako Takita, Wataru Kamitani, Haruyoshi Tomita

**Affiliations:** ^1^Department of Bacteriology, Graduate School of Medicine, Gunma University, Maebashi, Japan; ^2^Department of Infectious Diseases and Host Defense, Graduate School of Medicine, Gunma University, Maebashi, Japan; ^3^R&D Strategy & Planning Department, Kureha Corporation, Iwaki, Japan; ^4^Laboratory of Bacterial Drug Resistance, Graduate School of Medicine, Gunma University, Maebashi, Japan

**Keywords:** bacterial pathogenesis, antimicrobial resistance, porous carbon, virulence, enterohemorrhagic *Escherichia coli*, Shiga toxin, antimicrobial chemotherapy, Type III secretion system

## Abstract

Enterohemorrhagic *Escherichia coli* (EHEC) is one of the most common foodborne pathogens. However, no drug that prevents the severe complications caused by this bacterium has been approved yet. This study showed that a macroporous magnesium oxide (MgO)-templated carbon material (MgOC_150_) adsorbs Shiga toxins, and Type III secretory EspA/EspB proteins responsible for EHEC pathogenesis, and decreases the extracellular levels of these proteins. On the other hand, this material did not affect the growth of EHEC. *Citrobacter rodentium* traditionally used to estimate Type III secretion system-associated virulence in mice is highly virulent. The survival period of infected mice was prolonged when MgOC_150_ was administered. This adsorbent disturbed neither mammalian cells nor normal intestinal bacteria, such as *Enterococcus hirae*, *Lactobacillus acidophilus*, and *Lactobacillus casei*. In contrast, MgOC_150_ adsorbed antimicrobial agents, including β-lactams, quinolones, tetracyclines, and trimethoprim/sulfamethoxazole. However, fosfomycin and amikacin were not adsorbed. Thus, MgOC_150_ can be used with fosfomycin and amikacin to treat infections. MgOC_150_ is used for industrial purposes, such as an electrode catalyst, a bioelectrode, and enzyme immobilization. The study proposed another potential application of MgOC_150_, assisting anti-EHEC chemotherapy.

## Introduction

Shiga toxin-producing *Escherichia coli* (STEC) are a group of foodborne pathogens that can cause severe bloody diarrhea, hemorrhagic colitis, hemolytic uremic syndrome (HUS), which may lead to acute kidney failure, and neurological issues, such as acute encephalopathy (Tarr et al., 2005). Food poisoning caused by STEC is an infectious disease that affects more than 2.8 million people in 21 countries annually (Majowicz et al., 2014). STEC is known to produce two genetically distinct Shiga toxins named Stx1 and Stx2. Stx1 and Stx2 proteins bind to its receptor, globotriaosylceramide Gb3, localized on the host cell membrane, such as renal glomerular endothelial cells, resulting in cell death induction by inhibiting protein synthesis within host cells, which are closely associated with HUS development (Lingwood et al., 1987; Romer et al., 2007). The cytotoxicity of Shiga toxins depends on the Gb3 receptor because Gb3-deficient cells have very low susceptibility to these toxins (Shin et al., 2009). Shiga toxins also bind to the globotetraosylceramide Gb4 receptor to a lesser extent, and its receptor is involved in the induction of host cell death (Lingwood et al., 1987; Samuel et al., 1990). Enterohemorrhagic *E. coli* (EHEC) is the most important subgroup of STEC. In addition to Shiga toxins, EHEC produces effector proteins, which are responsible for the pathogenicity of this bacterium (Nataro and Kaper, 1998; Croxen and Finlay, 2010). Effector proteins are secreted *via* a protein transport machinery termed the Type III secretion system, and induce the formation of attaching and effacing (A/E) lesions in intestinal epithelial cells (Galan and Wolf-Watz, 2006). A/E lesions are characterized by the attachment of bacteria to the host cell membrane *via* the interaction of intimin and its receptor, the destruction of gut epithelial microvilli, and actin polymerization in the host cells (Kenny et al., 1997).

Antibiotics are commonly used to treat bacterial infections. However, the efficacy of antibiotic treatment for this infectious disease is controversial because *in vitro* experiments suggested that some antibiotics promote the release of Shiga toxins, which might increase the risk of HUS development (Wong et al., 2000; Safdar et al., 2002). Some alternative methods to prevent severe complications including HUS have been proposed, such as Gb3 analogs, recombinant antibodies that target Shiga toxins, effector proteins or intimin, and synthetic small organic molecules that inhibit Gb3 or Stx2 production (Nakao et al., 1999; Yamagami et al., 2001; Trachtman et al., 2003; Rasko et al., 2008; Yu et al., 2011; Ruano-Gallego et al., 2019). However, these molecules have not yet been approved as therapeutics.

Adsorbent is a term for materials that can trap certain chemical substances, and it is typically used to remove process contaminants in industries. Several adsorbents are also used as oral medicines. Colestyramine strong ion exchange resin is an approved medicine for the treatment of the hypercholesterolemia and the pruritus that often occurs during liver failure (Scaldaferri et al., 2013; Hegade et al., 2015). This drug acts as a bile acid sequestrant in the gastrointestinal tract. Activated charcoal is another type of adsorbent, and it can be used for the treatment of acute poisoning and the removal of bodily wastes associated with certain cardiovascular diseases (Zellner et al., 2019). AST120 (Kremezin) has been approved as an oral activated charcoal medicine to treat progressive chronic kidney disease as it removes the uremic toxin precursors produced by gut microorganisms (Niwa, 2011). In addition, a pilot study in clinical trial showed that a dietary supplement with an activated charcoal material reduced trimethyl amine concentration in urine from individuals with trimethylaminuria, and alleviated the symptoms of this disease (Yamazaki et al., 2004). However, no adsorbent medicine is currently approved for treatment of infectious diseases.

Porous carbon can adsorb non-polar organic molecules that fit its internal pore. Activated charcoal is the most well-known substrate of porous carbons, and it is made from carbon substrates, such as coals, coconut shells, and phenolic resins, and pores with various sizes can be generated in the activation process. Magnesium oxide (MgO)-templated carbon (MgOC) is another recently industrialized porous carbon. Its pore is produced by a method that is distinct from conventional activation (Inagaki et al., 2004). The pore template is formed by incorporating the MgO molecule generated during the pyrolysis of an Mg-containing organic substrate into a carbon matrix, and the pore is produced by removing the MgO molecule. This method enables the production of a more uniform size of pores than the activation method. Then, the resulting pores highly adsorb a targeted size molecule, although the adsorption of untargeted size molecules may be limited (Morishita et al., 2010).

This study aimed to establish a strategy to adsorb the Shiga toxins and Type III secretory proteins responsible for EHEC pathogenicity without disturbing host cells and beneficial bacteria in the host. In this study, we found that one MgOC material with an average pore size of 150 nm could adsorb both Shiga toxins and Type III secretory proteins produced by EHEC without impairing bacterial growth, including EHEC and several normal intestinal bacteria. Mice infected with bacteria exhibited an extended survival when MgOC was administered. Herein, we propose a potential option to treat EHEC/STEC infections.

## Materials and Methods

### Bacterial Strains, Host Cells, Culture Conditions, and Materials

EHEC O157:H7 Sakai, *Citrobacter. rodentium* DBS100, *Enterococcus. hirae* ATCC9790RF, *Lactobacillus. acidophilus* ATCC4356, and *Lactobacillus. casei* ATCC393 strains were used. EHEC and *C. rodentium* were cultured in Luria-Bertani (LB) medium unless otherwise indicated. *E. hirae* was cultured in Brain Heart Infusion (BHI) medium. *L. acidophilus* and *L. casei* were cultured in De Man, Rogosa, and Sharpe (MRS) medium. Bacteria were cultured in glass tubes at 37°C. Caco-2 (ATCC HTB-37) cells and Vero cells, and human kidney cells (HTB-44) were cultured in Dulbecco’s modified Eagle medium (DMEM) and Eagle’s minimal essential medium (EMEM), respectively containing 10% HyClone FetalClone III serum (HyClone Laboratories, Inc., Logan, UT, United States) at 37°C and in an atmosphere of 5% CO_2_. The MgOC material with a pore size of 150 nm, named MgOC_150_, was obtained from Toyo Tanso (Osaka, Japan). The activated charcoal material (made from coconut shell) was obtained from UES Co., Ltd. (Wakayama, Japan). The purified Shiga toxin protein and lysozyme were obtained from Nacalai Tesque (Kyoto, Japan).

### Shiga Toxin Assay

To estimate the amounts of Shiga toxins (Stx1 and Stx2), latex agglutination reagents (Denka Seiken Co. Ltd., Tokyo, Japan) were used. EHEC strains were cultured with shaking to the early stationary phase in Mueller–Hinton medium, and their cells and culture supernatants were separated by centrifugation at 13,000 g. Cell pellets were resuspended in EzBactYeastCrusher containing 60 mg/l lysozyme (ATTO, Tokyo, Japan) to extract intracellular proteins, and diluted into phosphate-buffered saline (PBS). These cell lysates (0.63 ng) and culture supernatants were serially diluted in 96-well round bottomed plates containing PBS, and an equal volume of latex suspension sensitized with the Stx1 or Stx2 antibody was then added. After incubation for 14 h at 4°C, titers were determined. The titers are presented as the reciprocal of the dilution of the last well before agglutinations were observed. To test the adsorption of Stx1 and Stx2 to MgOC_150_ and activated charcoal, the cell-free culture supernatant from EHEC was incubated with and without these materials for 2 h at 4°C. The cell-free culture supernatant was prepared by centrifugation at 13,000 g and passing through a membrane filter (pore size: 0.22 μm). After removal of porous materials by centrifugation at 15,000 *g*, Stx1 and Stx2 in the supernatant were assayed using latex agglutination reagents. To test the adsorption of purified Shiga toxin to MgOC_150_, the toxin (0.4 μg) was incubated with and without MgOC_150_ for 2 h at 4°C. Non-adsorbed toxin in the supernatant was quantified in a Bio-Rad protein assay according to the Bradford method (Bio-Rad Laboratories, Hercules, CA, United States). The cytotoxicity of Shiga toxins in Vero cells and HTB-44 was measured. Bacteria-free culture supernatants were 10-fold diluted into DMEM and EMEM, respectively containing, 10% HyClone FetalClone III serum, and added to cultured Vero and HTB-44 cells in 96-well plates. As a control, a 10-fold diluted Mueller–Hinton medium in DMEM and EMEM containing 10% HyClone FetalClone III serum was added to host cells. After incubation for 48 h, the cell viabilities were determined as previously described with CellTiter-Glo Luminescent Cell Viability Assay (Promega Corp., Madison, WI, United States; Hirakawa et al., 2021). Cell viabilities were represented as relative light units (RLUs) by their ratios (%) to the RLU of the control sample.

### Western Blotting

EHEC strains were cultured with shaking to an early stationary phase in Dulbecco’s modified Eagle medium (DMEM). Secreted proteins were precipitated from the supernatants using 10% trichloroacetic acid (TCA) and were dissolved in a Laemmli sample buffer (Bio-Rad Laboratories, Hercules, CA, United States). Bovine serum albumin (BSA) was used as a loading control and was added to the secreted protein samples prior to the precipitation with TCA. Intracellular proteins were resuspended in 50 mM phosphate buffer containing 8 M urea and then lysed by sonication. EspA and EspB were detected with their antisera, as previously described (Hirakawa et al., 2020a,b). To test the adsorption of EspA and EspB to MgOC_150_, cell-free culture supernatant prepared by centrifugation of EHEC culture at 13,000 g and passing through a membrane filter was incubated with and without MgOC_150_ for 2 h at 4°C. After removal of the MgOC_150_ material by centrifugation at 15,000 g, EspA and EspB in the supernatant was measured by western blotting.

### *Citrobacter rodentium* Infection in Mice

Three-week-old female C3H/HeJ mice were obtained from CLEA Japan (Tokyo, Japan).

MgOC_150_ was orally administered with a feed during the experiment as performed in previous studies used AST-120, an approved porous carbon medicine (Yang et al., 2017; Nakada et al., 2019). In those studies, 5 to 8% (w/w) of AST-120 was contained in a feed. For this reason, we supplied 7.5% (w/w) of MgOC_150_. The mice were housed for 7 days before infection (N = 5 control mice for non-infection and mice bled without MgOC_150_ for infection, N = 6 mice bled with MgOC_150_ for infection). *C. rodentium* DBS100 was cultured overnight in LB medium. Bacterial cells were resuspended in fresh LB medium at a concentration of 1 × 10^9^ CFU/ml, and 200 μl bacterial suspension (2 × 10^8^ CFU) was orally administered. As a control group, 200 μl bacteria-free LB medium was inoculated into mice. To measure the survival rates and body weight, mice were monitored daily for 21 days.

### Cytotoxicity Assays

To test the toxicity of MgOC_150_ in host cells, we used Caco-2 cells. MgOC_150_ was added to cultured Caco-2 cells. After incubation for 24 h, the cell viabilities were determined with CellTiter-Glo Luminescent Cell Viability Assay. The cell viabilities were represented as relative light units (RLUs) by their ratios (%) to RLU of the sample incubated without MgOC_150_.

### Adsorption Assays for Antimicrobial Agents

To estimate the capability of MgOC_150_ to adsorb antimicrobial agents, 1.25 mg aztreonam, ciprofloxacin, minocycline, trimethoprim, and sulfamethoxazole, 0.68 mg rifampicin, or 5 mg fosfomycin and amikacin in 5 ml aqueous solution were incubated with and without 30 mg MgOC_150_ for 2 h. Drug amounts were calculated as described previously (Hirakawa et al., 2020a).

### Statistical Analyses

We used the Gehan–Breslow–Wilcoxon tests for mouse survival experiments and the unpaired *t*-tests for mouse body weight measurement, cytotoxicity assays, and adsorption experiments, and then determined *p*-values using GraphPad Prism version 6.00.

## Results

### Macroporous Carbon MgOC_150_ Adsorbs Both Stx1 and Stx2 Shiga Toxins and Decreases the Extracellular Levels of These Toxins

**Figure 1:**
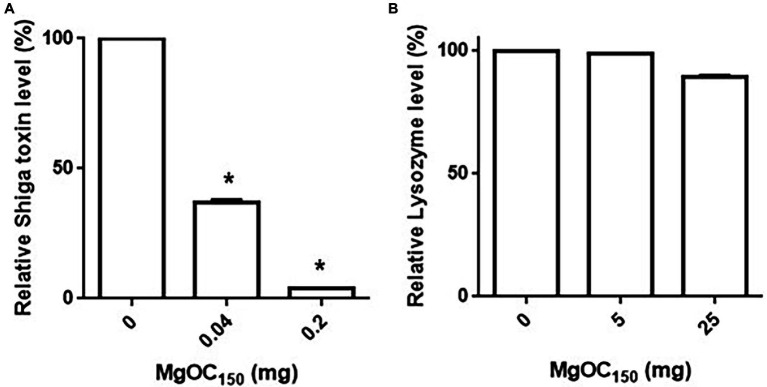
Adsorption of Shiga toxin **(A)** and lysozyme **(B)** by MgOC_150_. The Shiga toxin standard (0.4 μg) or lysozyme (50 μg) was incubated with and without MgOC_150_, and non-adsorbed proteins were measured in protein assay described in Materials and Methods. Protein levels are presented as the percentage of the value for samples after incubation with MgOC_150_ relative to that after incubation without MgOC_150_. Data plotted are the means from three independent experiments; error bars indicate the standard deviations. Asterisks denote significance for values (*p* < 0.05) of protein level after incubation with MgOC_150_ relative to that after incubation without MgOC_150_.

We aimed to find materials that adsorb proteins responsible for EHEC pathogenicity and attenuate the virulence of this bacterium. For this purpose, we used one MgOC material (MgOC_150_) with an average pore size of 150 nm because this size is predicted to highly adsorb protein molecules that are more than 50,000 Da (Funabashi et al., 2017). First, we tested the capability of MgOC_150_ to adsorb the Shiga toxins produced by EHEC. The bacteria-free supernatant from an EHEC culture was incubated with MgOC_150_, and Stx1 and Stx2 titers after MgOC_150_ removal were measured in latex agglutination assays. The agglutination titers of Stx1 and Stx2 from the EHEC supernatant were 64 and 128, respectively. However, no agglutination of Stx1 and Stx2 in the supernatant when incubated with 30 mg MgOC_150_ was observed (Table 1). To compare the capability of MgOC_150_ with ordinal activated charcoal, the bacteria-free supernatant was incubated with 30 mg activated charcoal made from coconut shells. The agglutination titers of Stx1 and Stx2 were then measured. These titers were the same as those in supernatants incubated without the activated charcoal (Table 1). These observations indicated that MgOC_150_, not activated charcoal, highly adsorbs Shiga toxins. To estimate the adsorption affinity of MgOC_150_ to Shiga toxins, a purified Shiga toxin standard was incubated with different amounts of MgOC_150_. More than 60% of the toxin (0.4 μg) in a solution was adsorbed when incubated with at least 0.04 mg of MgOC_150_, and more than 95% of the toxin was adsorbed when incubated with 0.2 mg of MgOC_150_ (Figure 1A). We also tested the ability of MgOC_150_ to adsorb lysozyme, a small-sized protein. Its predicted molecular size is approximately 14,300 Da thus, the ability of MgOC_150_ to adsorb this protein molecule may be low. As predicted, more than 90% of the lysozyme protein (50 μg) remained in a solution even after being incubated with 25 mg of MgOC_150_ (Figure 1B). We next measured the levels of Shiga toxins in bacterial cultures. EHEC was cultured with MgOC_150_, and agglutination titers were then determined. No agglutinations of Stx1 and Stx2 in the EHEC supernatant cultured with 30 mg MgOC_150_ were observed, whereas there was no apparent difference in these titers between the cell lysates from strains cultured with and without MgOC_150_ (Table 2). Bacterial colony-forming units (CFUs) were essentially the same when cultured with and without MgOC_150_ (data not shown). Therefore, MgOC_150_ could reduce extracellular Stx1 and Stx2 levels in EHEC without suppressing bacterial growth. These results indicated that MgOC_150_ adsorbs the Stx1 and Stx2 secreted by EHEC and decreases the extracellular Stx1 and Stx2 levels but does not impair Shiga toxin production and EHEC growth.

**Table 1:** Shiga toxin titers after adsorption by porous carbons.

Porous carbon materials*	Shiga toxin titers	
	Stx1	Stx2
None	64	128
MgOC_150_	<2	<2
Activated charcoal	64	128

* *30 mg of MgOC_150_ or ordinal activated charcoal (from coconuts shells) was added into 5 ml of the bacteria-free supernatant and incubated. Shiga toxin titers were determined after removal of MgOC_150_ and the activated charcoal material*.

**Table 2:** Shiga toxin titers in EHEC culture supernatant and cell lysate.

MgOC_150_	Shiga toxin titers in supernatant*
	Stx1		Stx2
0 mg	64	128
5 mg	64	64
10 mg	16	32
30 mg	<2	<2
*Shiga toxin titers in cell lysate* *
0 mg	64	16
30 mg	64	16

* *EHEC was cultured with and without MgOC_150_ in 5 ml medium. After culture, supernatant and bacterial cells were separated. Shiga toxin titers in each fraction were determined*.

**Figure 2:**
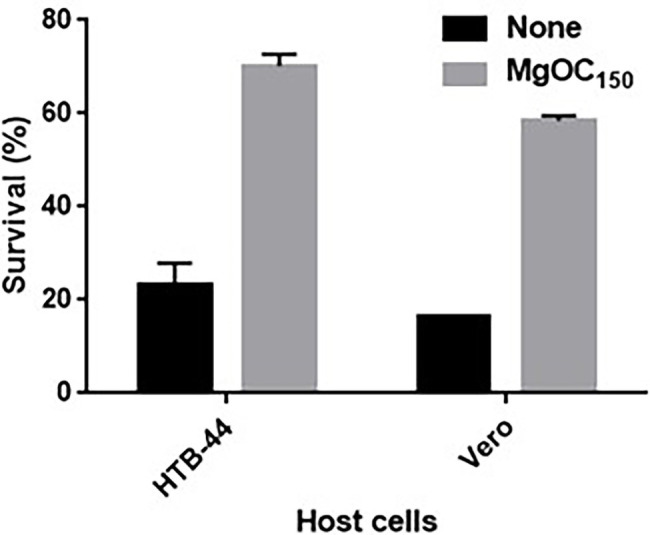
Survival of HTB-44 and Vero cells after incubation with and without EHEC supernatant cultured with and without MgOC_150_. Survival rates are presented as the percentages of the RLU value for cells after incubation with each supernatant relative to that after incubation without supernatant. Data are the means of two biological replicates. Error bars indicate the ranges. Experiments were repeated twice, and similar results were obtained.

Shiga toxins cause damage to human renal cells (Kiyokawa et al., 1998). We tested whether the removal of Shiga toxins by MgOC_150_ treatment reduces toxicity to human HTB-44 renal cells. The addition of a bacteria-free supernatant from an EHEC culture killed approximately 80% of HTB-44 cells, whereas 70% of the host cells survived when incubated with EHEC supernatants cultured with 30 mg MgOC_150_ (Figure 2). The cytotoxicity of Shiga toxins is also commonly evaluated in Vero cells as alternative cells (Konowalchuk et al., 1977). The toxicity of EHEC supernatants cultured with and without MgOC_150_ to Vero cells was also examined. The addition of the supernatant killed approximately 85% of Vero cells. Similar to HTB-44 cells, the cytotoxicity of the supernatant cultured with 30 mg MgOC_150_ was modest (58% of the cells survived; Figure 2). These results suggested that MgOC_150_ decreases Shiga toxin-associated cytotoxicity.

### MgOC_150_ Also Adsorbs Type III Secretory Proteins, Including EspA and EspB, and Decreases the Levels of These Proteins

**Figure 3:**
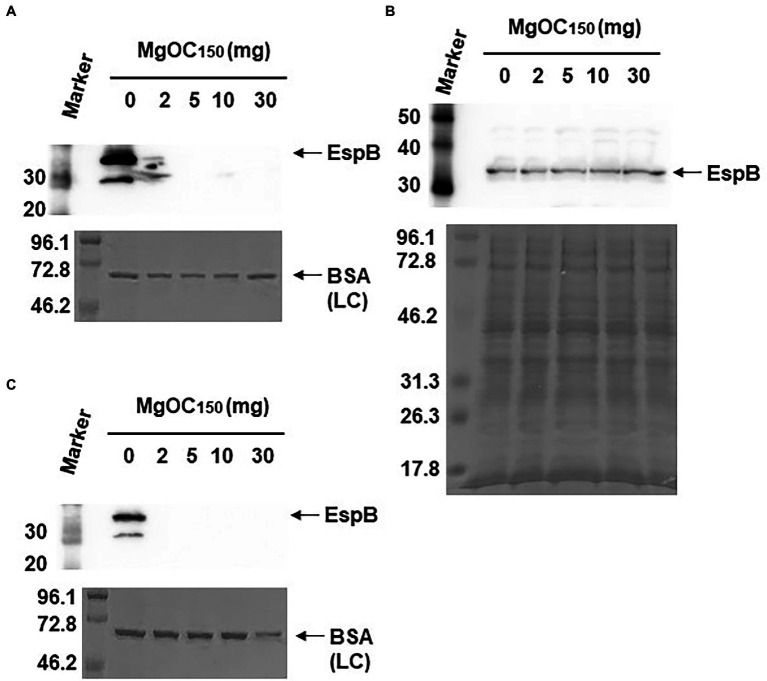
Determination of EspB levels. **(A)** EspB levels in the EHEC supernatant cultured with and without MgOC_150_. **(B)** EspB levels in EHEC whole-cell extracts cultured with and without MgOC_150_. **(C)** The EHEC supernatant was incubated with and without MgOC_150_ for 2 h, and EspB levels were estimated after MgOC_150_ removal. Proteins including EspB were separated by SDS-PAGE. EspB was visualized by Western blotting with EspB antiserum. For loading control (LC), BSA was visualized by CBB stain. Locations of molecular mass standards (in kilodaltons) are shown on the left.

**Figure 4:**
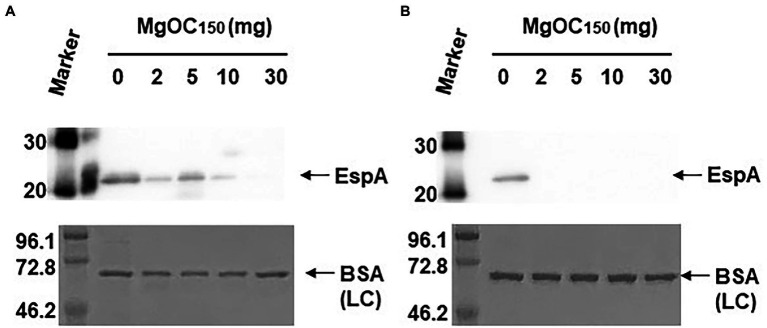
Determination of EspA levels. **(A)** EspA levels in the EHEC supernatant cultured with and without MgOC_150_. **(B)** The EHEC supernatant was incubated with and without MgOC_150_ for 2 h, and EspA levels were estimated after MgOC_150_ removal. Proteins including EspA were separated by SDS-PAGE. EspA was visualized by Western blotting with EspA antiserum. For loading control (LC), BSA was visualized by CBB stain. Locations of molecular mass standards (in kilodaltons) are shown on the left.

Type III secretory proteins are the other subset of proteins required for EHEC virulence. EspB is a member of these proteins, and it is required to translocate other effector proteins into host epithelial cells. This protein also has an effector activity (Taylor et al., 1999; Kodama et al., 2002). The EspB level in EHEC supernatants and cell extracts cultured with and without MgOC_150_ was measured. The addition of at least 2 mg MgOC_150_ reduced the EspB level in the supernatant (Figure 3A). In contrast, the EspB level in the cell extract was not reduced even when 30 mg MgOC_150_ was present (Figure 3B). The capability of MgOC_150_ to adsorb the EspB protein was also assessed. The EHEC supernatant was incubated with MgOC_150_, and the EspB level in the supernatant was measured after MgOC_150_ removal. No residual EspB protein was observed in the supernatant after incubation with at least 2 mg MgOC_150_ (Figure 3C). In addition to EspB, the adsorption and extracellular accumulation of EspA another protein secreted *via* the Type III secretion system were examined. MgOC_150_ also adsorbed the EspA protein and reduced its extracellular level (Figures 4A,B).

### MgOC_150_ Attenuates the Virulence of *Citrobacter Rodentium* in Mice

**Figure 5:**
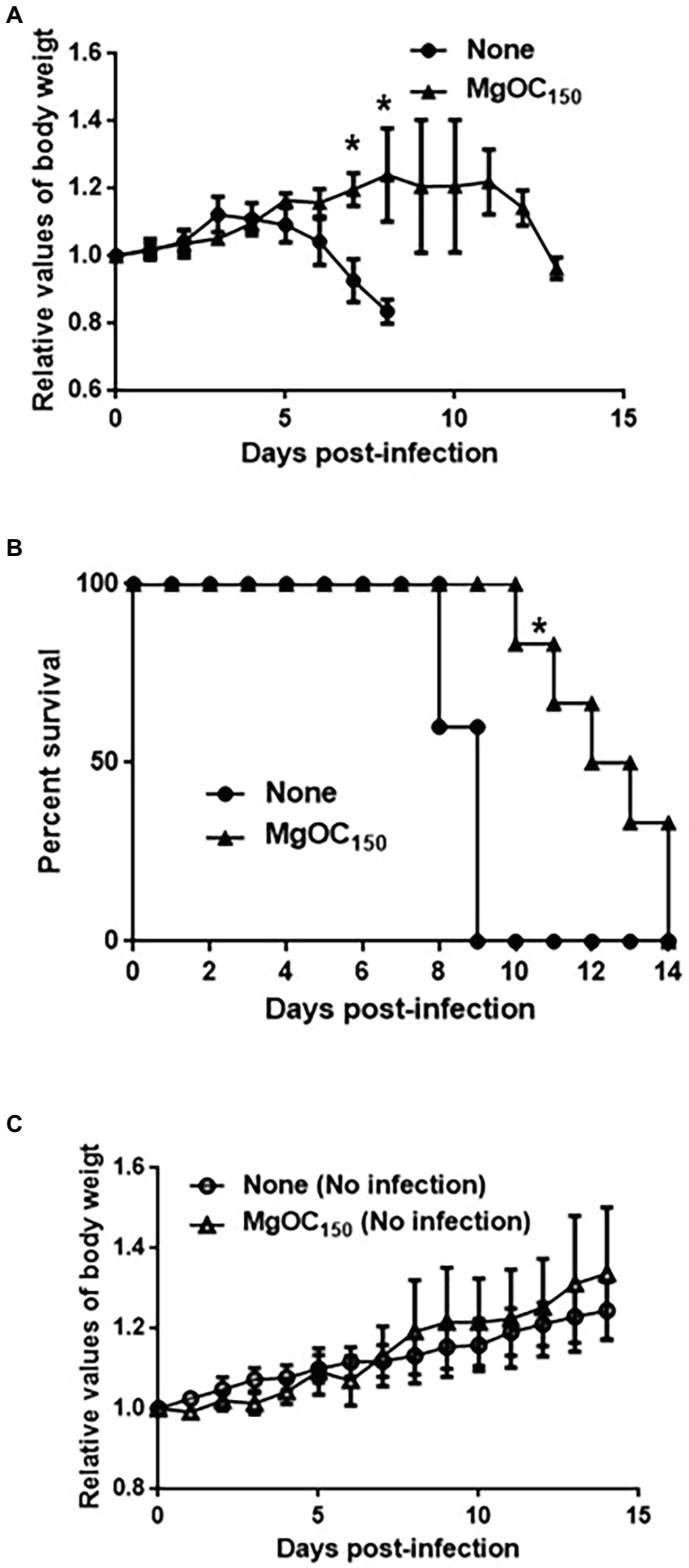
Virulence of *C. rodentium* in C3H/HeJ mice after MgOC_150_ administration. **(A)** Change in the body weight of C3H/HeJ mice infected with the DBS100 strain. The connecting lines denote the means, and the error bars denote the standard deviations. **(B)** Survival of C3H/HeJ mice infected with the DBS100 strain. **(C)** Change in the body weight of C3H/HeJ control mice. The connecting lines denote the means, and the error bars denote the standard deviations. Mice (*N* = 5 mice bled without MgOC_150_ for infection, *N* = 6 mice bled with MgOC_150_ for infection, *N* = 5 control mice bled without MgOC_150_ for non-infection, and *N* = 5 control mice bled with MgOC_150_ for non-infection) were monitored daily. Asterisks denote the significances (*p* < 0.05) of survival rate and body weight of mice administrated with MgOC_150_ relative to those of mice administrated without MgOC_150_.

To assess the *in vivo* effectiveness of MgOC_150_ that attenuates bacterial virulence, a murine intestinal infection model with *C. rodentium* was used. *C. rodentium* is a natural pathogen of mice, and it causes typical diarrheal symptoms (Mundy et al., 2005). This pathogen produces one subset of orthologous Type III secretory proteins including EspA and EspB, but does not have genes that encode Shiga toxins. For this reason, *C. rodentium* is used to evaluate Type III secretion system-associated virulence in mice. To treat mice with MgOC_150,_ it was administered with their feed. The mice did not adequately consume the MgOC_150_-containing diet on the first few days, but they consumed the diet normally after that. Thus, the mice were pretreated with MgOC_150_ for 7 days before infection and the treatment was maintained until the end of experiments. The *C. rodentium* DBS100 strain that is highly virulent in C3H/HeJ mice was used. A decrease in body weight was observed after 4 days of infection in non-treated mice, and all mice died within 9 days post-infection (Figures 5A,B). When MgOC_150_ was administered with the feed, the mice infected with *C. rodentium* exhibited no body weight decrease until 11 days post-infection, and mice survived significantly longer (Figures 5A,B). The median duration of survival could be prolonged up to 12.5 days post-infection. These results suggested that MgOC_150_ administration reduces the virulence of *C. rodentium* in mice. No significant difference in weight gain between non-infected control mice fed with a regular diet and a MgOC_150_-containing diet was observed (Figure 5C).

### MgOC_150_ Does Not Disturb Host Cells and Some Species of the Normal Intestinal Flora, Such as *Enterococcus* and *Lactobacillus*

**Figure 6:**
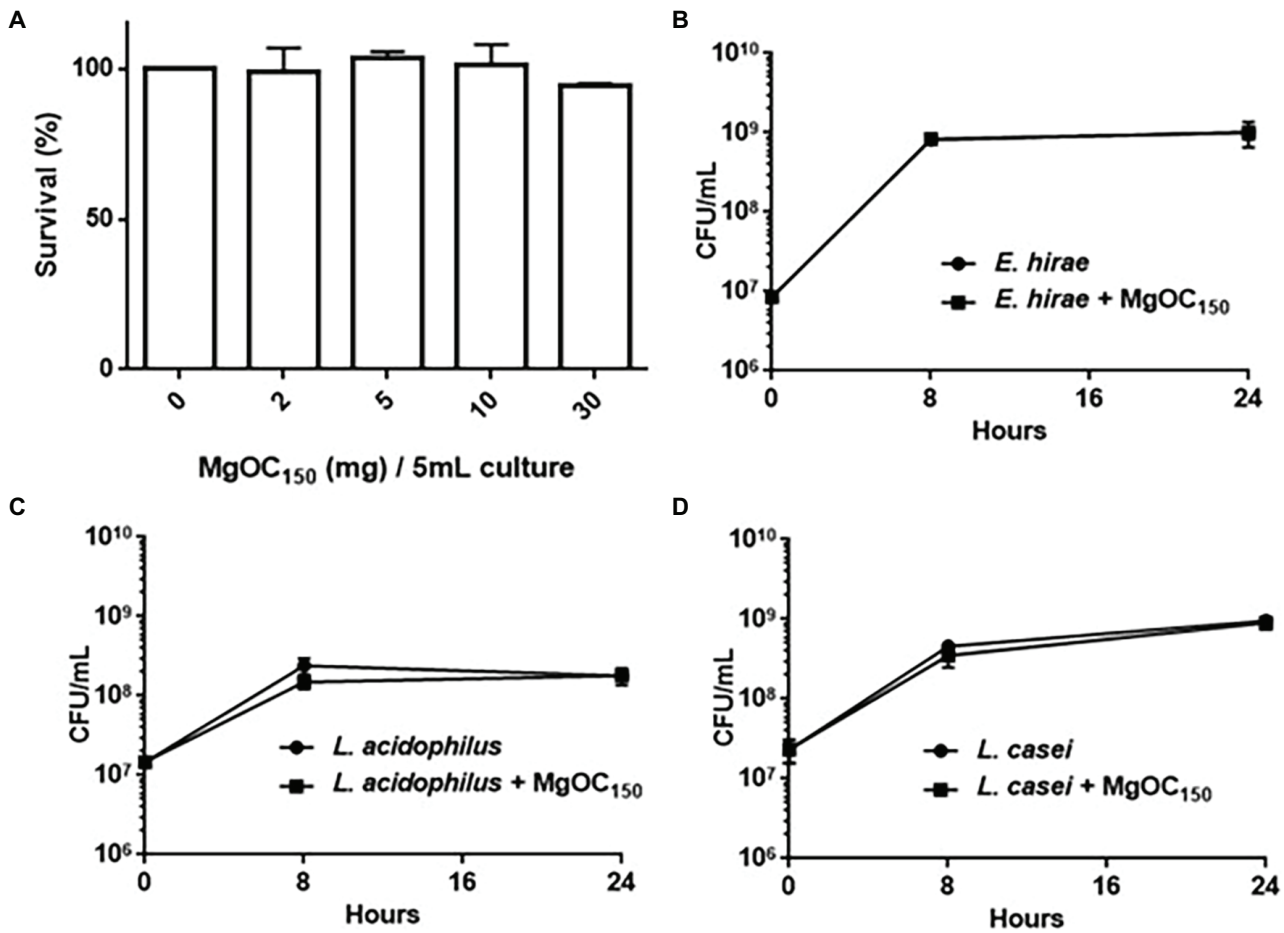
Toxicity of MgOC_150_ in host cells and normal intestinal flora. **(A)** Survival of Caco-2 cells after incubation with without MgOC_150_. MgOC_150_ was added into 150 μl Caco-2 cell cultures. Amounts (mg) of MgOC_150_ on *x*-axis were represented as those corresponding to 5 ml cultures. The survival rates are presented as the percentage of the RLU value for the cells after incubation with MgOC_150_ relative to that after incubation without MgOC_150_. Growth of several normal intestinal flora, *E. hirae* **(B)**, *L. acidophilus* **(C)**, and *L. casei* **(D)** cultured with and without MgOC_150._ All strains were anaerobically cultured with and without 30 mg MgOC_150_. Bacterial growth was estimated by measuring the CFUs.

We used Caco-2 cells to test the cytotoxicity of MgOC_150_ in human intestinal epithelial cells. The host cells were incubated with MgOC_150_ for 24 h. MgOC_150_ did not exhibit cytotoxicity because no significant reduction of viable cell numbers was observed after incubation with MgOC_150_ (Figure 6A). When MgOC_150_ is orally administered, it may disturb normal bacterial flora in the intestinal tract by adsorbing some beneficial compounds and/or bacteria. To test this hypothesis, the growth of *Enterococcus hirae*, *Lactobacillus acidophilus*, and *Lactobacillus casei*, which are commonly isolated from the normal intestine, was examined after being cultured with and without MgOC_150_ for 8 and 24 h_._ When the bacterial strains were cultured without MgOC_150_, the CFUs of *E. hirae*, *L. acidophilus*, and *L. casei* reached 8.2 × 10^8^, 2.4 × 10^8,^ and 4.6 × 10^8^ after 8 h, and 1.0 × 10^9^, 1.8 × 10^8^ and 9.6 × 10^8^ after 24 h, respectively (Figures 6B–D). The CFUs of these bacteria reached similar values even when cultured with 30 mg MgOC_150_ (8.0 × 10^8^, 1.5 × 10^8^, and 3.5 × 10^8^ after 8 h, and 1.0 × 10^9^, 1.8 × 10^8^, and 9.0 × 10^8^ after 24 h, respectively). These results indicated that MgOC_150_ does not disturb *E. hirae*, *L. acidophilus*, and *L. casei*.

### MgOC_150_ Does Not Adsorb Fosfomycin and Amikacin, and these Drugs Are Still Active in the Presence of MgOC_150_

**Figure 7:**
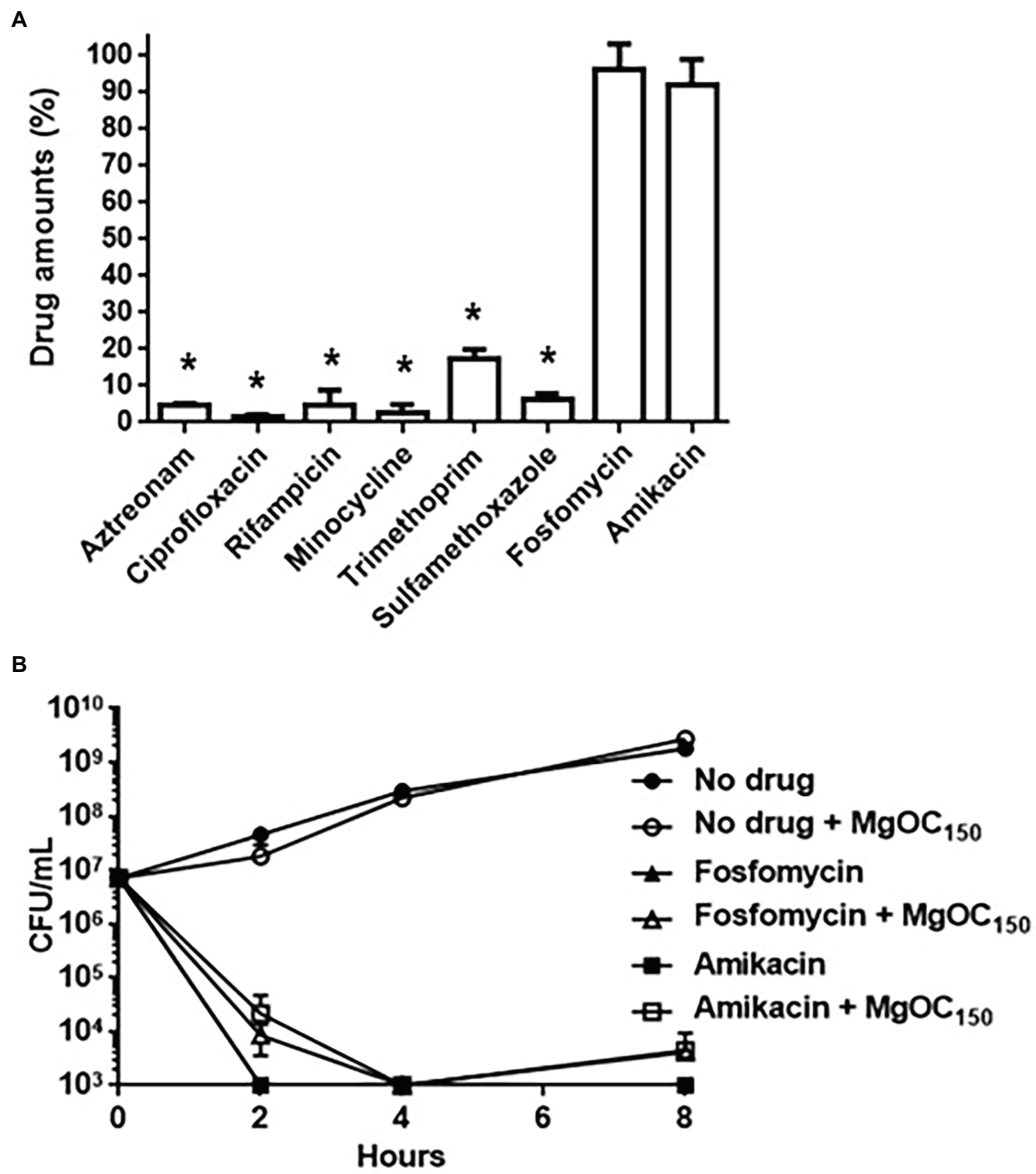
Antimicrobial activities of indicated drugs in the presence of by MgOC_150_. **(A)** Drug adsorption by MgOC_150_. Each drug was incubated in an aqueous solution with and without 30 mg MgOC_150_ for 2 h. The *y* axis shows the percent of drug amount (mg) after incubation with MgOC_150_ relative to the drug amount (mg) after incubation without MgOC_150_. **(B)** Growth of the EHEC strain when cultured with or without indicated drugs in the presence and absence of 30 mg MgOC_150_. Data plotted are the means from three independent experiments; error bars indicate the standard deviations. Asterisks denote significance for values (*p* < 0.05) of drug amount and CFU (colony-forming unit) after incubation with MgOC_150_ relative to that after incubation without MgOC_150_.

MgOC_150_ may adsorb some antimicrobial agents. This property may impair a combination therapy that uses MgOC_150_ and an antimicrobial agent. We tested whether MgOC_150_ adsorbs antimicrobial agents that are generally active in EHEC. Each antimicrobial agent was incubated with and without 30 mg MgOC_150_ for 2 h and the amount of each agent in the supernatant was measured after MgOC_150_ removal. Undesirably, aztreonam, ciprofloxacin, rifampicin, minocycline, trimethoprim, and sulfamethoxazole were highly adsorbed by MgOC_150_, as these amounts after incubation with MgOC_150_ were lower than 20% compared to the MgOC_150_-free control (Figure 7A). In contrast, fosfomycin and amikacin retained more than 80% even after incubation with MgOC_150_ (Figure 7A). The *in vitro* activities of fosfomycin and amikacin in EHEC were examined when cultured with MgOC_150_. Bacterial cell deaths were observed as a significant reduction in bacterial CFUs was found when cultured with 32 μg/ml fosfomycin and 64 μg/ml amikacin. Similar reductions in CFUs were also observed even when MgOC_150_ was present (Figure 7B). Therefore, MgOC150 does not impair the antimicrobial activities of fosfomycin and aminoglycosides, such as amikacin.

## Discussion

Some molecules that attenuate the virulence of EHEC have been proposed [recently reviewed in Muhlen and Dersch, 2020]. However, no clinically approved method that absolutely prevents severe complications, including HUS, has been established yet. This study showed that a macroporous MgOC particle MgOC_150_ adsorbed both Shiga toxins and Type III secretory proteins EspA/EspB and reduced the extracellular levels of these proteins responsible for EHEC pathogenesis (Tables 1, 2; Figures 1-4), whereas it impaired neither host nor several members of normal intestinal flora (Figures 5, 6). MgOC_150_ is used for industrial purposes, such as an electrode catalyst, a bioelectrode, and enzyme immobilization (Tsujimura et al., 2014; Funabashi et al., 2017; Mazurenko et al., 2018). Herein, we propose another benefit of MgOC_150_ for the development of anti-EHEC/STEC chemotherapy. Previous *in vitro* research has shown that an activated charcoal material adsorbed the Shiga toxins produced by EHEC (Naka et al., 2001). However, it also undesirably adsorbed beneficial intestinal bacterial cells. This study used another activated charcoal material that showed no adsorption of Shiga toxins. We suggest that MgOC_150_ is superior to these conventional activated charcoals.

Although MgOC_150_ adsorbs certain molecules in a non-specific manner, it is predicted to highly adsorb protein molecules that are more than 50,000 Da (Funabashi et al., 2017). Shiga toxins are released into the extracellular space as free protein complexes consisting of a monomer A subunit protein and pentamer B subunit proteins (Le Nours et al., 2013). Their whole molecular sizes are estimated at 70,000 Da. Therefore, the effective adsorption of Shiga toxins by MgOC_150_ is reasonable while the ability to adsorb lysozyme, a small-sized protein is relatively low. In contrast, the monomer sizes of EspA and EspB are approximately 21,000 and 33,000 Da, respectively. These are much smaller than the average pore sizes in MgOC_150_. EspA and EspB proteins form heteroprotein complexes with other protein members composing the Type III secretion system and plasma membrane proteins of host cells when EHEC attaches to the host cells. EspA forms a polymeric filamentous structure and builds a bridge between bacteria and host cell surfaces when it attaches to the EscF needle protein (Wilson et al., 2001). EspB is delivered *via* EspA and then forms a pore structure with the EspD protein on the host cell membrane (Ide et al., 2001). This protein also enters the host cells, where it binds to several host proteins, such as α-catenin and myosin (Kodama et al., 2002; Iizumi et al., 2007). In the *in vivo* experiment used the alternative *C. rodentium* and mice, MgOC_150_ attenuated the virulence of this bacterium in mice (Figure 5). *C. rodentium* is commonly used to evaluate virulence associated with the Type III secretion system in the intestine of mice. Therefore, we believe that EspA and EspB proteins are adsorbed by MgOC_150_ *in vivo*. This *in vitro* study showed the adsorption of these proteins in the host cell-free bacterial supernatant (Figures 3, 4). The oligomeric states of EspA and EspB proteins in the bacterial supernatant are unknown when host cells are absent. Some studies showed that the EspB protein could bind to EspA and EspD proteins in a solution even when host cells are absent (Hartland et al., 2000; Ide et al., 2001; Yip et al., 2005). If EspA and EspB form a complex with EspD, this protein complex may fit the pores of MgOC_150_.

EspA and EspB expression is induced by the indole produced by some of enteric bacteria (Hirakawa et al., 2009). We previously found that AST-120, an oral adsorbent, eliminated the effect by adsorbing the indole molecule (Hirakawa et al., 2020a). MgOC_150_ may not eliminate the indole effect because addition of MgOC_150_ decreased neither EspA nor EspB expression. In contrast to MgOC_150_, the average pore size of AST-120 is approximately 2 nm, and this pore size is suitable for highly adsorbing the indole molecule, although it may be too small to adsorb EspA and EspB proteins. This may support the difference in adsorption capabilities of MgOC_150_ and AST-120 for the indole molecule.

However, MgOC_150_ adsorbed antimicrobial agents such β-lactams and quinolones, which are commonly used to treat bacterial infections, despite the fact that the sizes of these antimicrobial molecules are much smaller than the pore size of MgOC_150_ (Figure 7A). The pore of MgOC_150_ is produced from an Mg-containing template substrate. If some hydroxyl groups derived from this substrate molecule remain even after the pyrolysis process, a significant amount of a small antimicrobial compound may be bound by this hydroxyl group. Therefore, improving the method of pore production may be important to minimize the adsorption of small antimicrobial compounds. In contrast, the adsorption capability for fosfomycin and aminoglycosides was relatively low (Figure 7A). For this reason, MgOC_150_ did not impair activities of these drugs (Figure 7B). The use of antibiotics is controversial for the treatment of STEC infections because the release of Shiga toxins is promoted by antibiotics-induced bacterial cell lysis (Wong et al., 2000; Safdar et al., 2002). Antibiotics could be effective for non-STEC, such as enteropathogenic *E. coli*, which do not produce Shiga toxins, but still produce Type III secretory proteins. MgOC_150_ may offer a benefit by assisting conventional fosfomycin and aminoglycoside therapy to treat non-STEC infections.

We note that our *in vivo* study has certain limitations. The effectiveness of MgOC_150_ to attenuate the cytotoxicity associated with Shiga toxins was validated *ex vivo* by using Vero and human kidney epithelial cells (Figure 2). However, we used the *C. rodentium* DBS100 strain, which does not produce Shiga toxins, for the *in vivo* experiment (Figure 5). Mice were pretreated with MgOC_150_ before infection for the technical reason mentioned above. Therefore, the results of our *in vivo* experiment show a prophylactic value for this adsorbent, although the exact mechanism of this *in vivo* prophylaxis has not been discerned. The therapeutic utility of MgOC_150_ and *in vivo* adsorption of Shiga toxins need to be addressed in the future. In addition, the safety of this adsorbent must be extensively validated for medical applications. We provided evidence that the administration of MgOC_150_ impairs neither mice nor human intestinal epithelial cells (Figures 5, 6). Although the current study showed that MgOC_150_ does not disturb several species of normal intestinal flora, a comprehensive study would be necessary to fully understand the impact on gut microbiome. However, the idea of using microporous adsorbent may open the door to develop an anti-EHEC/STEC therapy.

## Data Availability Statement

The original contributions presented in the study are included in the article/supplementary material, further inquiries can be directed to the corresponding author.

## Ethics Statement

The animal study was reviewed and approved by the Committee of Experimental Animal Research of Gunma University (The approval number: 19-094).

## Author Contributions

HH, KS, MU, WK, and HT designed the research and wrote the manuscript. HH, KS, WK, and HT analyzed the data. HH, KS, and AT performed the experiments. All authors contributed to the article and approved the submitted version.

## Funding

This study was kindly supported by JSPS KAKENHI “Grant-in-Aid for Scientific Research (C)”; grant number 19K07533 (HH), and “Grant-in-Aid for Scientific Research (B)” grant number 22H02864 (HH and KS), the Takeda Science Foundation (HH), the Ohyama Health Foundation (HH), Japan Agency Research and development [AMED]; grant numbers 22fk0108604h0901, and 22wm0225008h0202 (HT), and The Ministry of Health Labour Welfare “Health Labour Sciences Research Grant”; grant number 21KA1004 (HT).

## Conflict of Interest

MU, belongs to a commercial company, Kureha corp. This author contributed to the study design and data interpretation, but did not directly participate in data collection.

The remaining authors declare that the research was conducted in the absence of any commercial or financial relationships that could be construed as a potential conflict of interest.

## Publisher’s Note

All claims expressed in this article are solely those of the authors and do not necessarily represent those of their affiliated organizations, or those of the publisher, the editors and the reviewers. Any product that may be evaluated in this article, or claim that may be made by its manufacturer, is not guaranteed or endorsed by the publisher.
